# Förnuft och känsla – Kunskapsbruk hos gårdagens förbudskritiker och dagens alkoholliberaler

**DOI:** 10.1177/14550725211072631

**Published:** 2022-03-07

**Authors:** Katarina Winter, Johan Edman

**Affiliations:** 398666 Stockholm University, Stockholm, Sweden; 398666 Stockholm University, Stockholm, Sweden

**Keywords:** public health policy, alcohol policy, knowledge production, think tanks, timbro, the prohibition battle, science and technology studies

## Abstract

**Aim:** The aim is to study non-governmental actors’ production and use of alcohol policy knowledge in the early 20th and the 21st century respectively, by analyzing their main arguments, knowledge substantiation and their overarching discursive legitimacy. **Design:** The first impact focuses on prohibitionist-critical actors’ engagement against the alcohol ban in the years 1916–1922. The second impact focuses on the Swedish think tank Timbro’s engagement in alcohol policy in the years 2012–2020. The analysis of the two empirical cases was based on an open coding strategy with a focus on what type of knowledge claims that were made and how which reasoning was put forward in relation to these. **Results:** Great similarities are distinguished between the two time periods. Alcohol is an issue of freedom and at the same time a threat of crucial importance for the future society. The arguments are supported by historical, international, media and scientific evidence. The biggest difference lies in the legitimization of the argumentation. In the early 20th century this is rooted in democracy and the will of the people while the arguments of the 21st century are rooted in public health and governmentally sanctioned knowledge. **Conclusion:** The knowledge processes are explored as matters of political appropriation that takes place through processes of directing and stealing the spotlight. These processes show how the aspiring democracy and the existing public health policy respectively are productive preconditions for what kind of knowledge that can be brought forward. This enables a renegotiation regarding what democracy and public health policy can involve.

## Inledning

Albert Engströms berömda sentens om vad kräftor kräver för drycker är troligen det budskap som flest människor förknippar med 1922 års folkomröstning om ett alkoholförbud. Det är text och bild i skön harmoni men frågan är om inte kräftaffischens ikoniska status mer beror på den kände upphovsmannen och på att budskapet är så befängt. Vem låter kräftor bestämma hur man ska rösta i en folkomröstning?

Kanske var detta gastronomiska och emotionella argument ändå vad som behövdes vid en tid då flera länder på kort tid hade infört olika varianter av alkoholförbud, bland dem tre nordiska grannländer. Den svenska kräftan krävde inte så mycket det svenska brännvinet som att brännvinet krävde kräftan. Alkoholfrågan hade formulerats som ett problem i Sverige och övriga västvärlden och som problemkonstruktörer ägde de nykterhetsvänliga krafterna den frågan. Forskning, utredningar, lagförslag och nykterhetsrörelsens egna utspel bidrog till en massiv kunskapsproduktion som pekade ut problemets karaktär, orsaker och lösningar. Förbudsmotståndarnas ingång i denna fråga var med nödvändighet helt annorlunda; de hade att bestrida förekomsten av ett problem som de själva inte uppfattade som ett problem. Mot den samlade nykterhetsrörelsen och den statliga kunskapsproduktionen, främst representerad av nykterhetskommitténs 18 betänkanden publicerade åren 1913–1921, ställde förbudsmotståndarna en kräftskiva. Det var inga bra odds.

Ändå vann den så kallade nej-sidan folkomröstningen.^i^ Det personliga intresset hos folkflertalet av att få fortsätta konsumera alkohol ska inte underskattas men där fanns också en argumentation mot ett alkoholförbud som sträckte sig längre än det så kallade kräftargumentet. Jämte den något vulgära *N.E.J.-Centralen* (vilka låg bakom flera kända affischer, däribland Albert Engströms) bedrevs förbudsmotståndet av sammanslutningar med en något mer sofistikerad argumentation, en sorts utomstatliga påverkansorganisationer och aktörer som vi idag närmast skulle beskriva som lobbyorganisationer eller tankesmedjor. Idag har den typen av organisationer ökat avsevärt i antal. Även om långt ifrån alla är engagerade i alkoholfrågan är detta något som utmärker Sveriges största tankesmedja, Timbro.

I den här artikeln utforskar vi två olika varianter av dessa utomstatliga kunskapsaktörer.^1^ Det första nedslaget fokuserar på förbudskritiska aktörers engagemang mot alkoholförbudet åren 1916–1922. Det andra nedslaget fokuserar på tankesmedjan Timbros alkoholpolitiska engagemang åren 2012–2020. Syftet är att analysera utomstatliga aktörers alkoholpolitiska kunskapsbruk i början av 1900-talet respektive 2000-talet genom att studera deras huvudargument, kunskapsunderbyggnad och övergripande diskursiva legitimering (Johansen et al. 2021).

## Kunskapsproduktion och policypåverkan

Vårt intresse för kunskapsbruk inom det alkoholpolitiska fältet av flera skäl. För det första finns det en mängd konkurrerande kunskapsanspråk på detta fält, där det blir tydligt att förslag för eller mot förbud/restriktioner respektive liberaliseringar kan beskrivas som lika viktiga för ett gott samhälle. Det handlar om vilka aspekter som alkoholpolitiken bör prioritera där exempelvis ett fokus på individens frihet eller hälsa medför olika policyimplikationer. För det andra kan en undersökning av kunskapslegitimering inom detta fält belysa den bredare frågan om relationen mellan verklighetsbeskrivning och policyåtgärder. I en tid då det ropas på evidensbaserad politik inom en mängd områden kan man å ena sidan få intrycket att detta är något nytt och å andra sidan att det skulle vara en garant för rationell politik. Vi hoppas kunna visa att det varken är nytt eller någon rimlig modell för postideologisk rationalitet.

Historiska undersökningar om den svenska välfärdsstaten brukar framhålla den mer än hundraåriga relationen mellan beslutsfattare och experter (Benner & Widmalm, 2011; Heclo, 1974; Hirdman, 1989; Lundqvist & Petersen, 2010). Det svenska utredningsväsendets ses som ett exempel på hur politik och forskning tillsammans har skapat det moderna Sverige; det talas till och med om Sverige som en vetenskaplig stat (Lundin, 2010). Det finns dock en inneboende konflikt här då både politik och vetenskap präglas av såväl interna som relationella motstridigheter och intressen. Materialet i vår undersökning är exempel på detta.

En bred definition av policypåverkande kunskapsaktörer förlägger dock fenomenet långt tillbaka i historien och rymmer såväl en Machiavelli som en Richelieu och en Clausewitz. Vårt syfte är smalare och tar sikte på den kunskapslegitimering som nyttjat och nyttjats av politiken i en demokratisk era, en i Sverige sekellång period som har krävt särskilda instrument för politiskt maktutövande. Även om merparten av det undersökta materialet i vårt första exempel härrör från åren strax innan den allmänna rösträttens förverkligande 1921 så var denna en snart realiserad verklighet att förhålla sig till, något som också tydligt syns i det undersökta materialet. Att kunna appellera till det bästa för folket i en tid när också folket har makten att påverka politiken på området är något som förenar våra två empiriska exempel och som tydligt har påverkat argumentationslinjerna.

Den moderna formen av påverkansorganisationer och vad som senare blev känt som tankesmedjor daterar sig också till ungefär denna tid. Vi finner dem i USA för drygt 100 år sedan (Teitz, 2009), men deras ökande betydelse och numerära tillväxt daterar sig främst till 1970-talet (Rich, 2001). De kan definieras som agendasättande (Rich, 2005) eller policyorienterade forskningskonstellationer (Medvetz, 2012; Smith, 1991), även om vi i vår undersökning också ser mer lösa konstellationer av kunskapslegitimerade policyanspråk. Det handlar både om vad Hawkins och Parkhurst (2016, s. 577) beskriver som “forskningsfokuserade institutioners (universitet, forskningsinstitut, tankesmedjor och NGO:s)” ansvar för att tillföra evidens till policyprocesser och om engagerade forskare och medborgare med specifik kunskap om avgränsade frågor. Vad som förenar dem är att detta är aktörer som staten måste förhålla sig till (Rich, 2005); detta är åtminstone deras utgångspunkt i påverkansarbetet.

Påverkan kan bestå i både forskningsrapporter och andra aktiviteter, där forskningen exempelvis kan vara beställd av industriaktörer (Stone, 1996), men också i att kommunicera alternativa kunskapsunderlag för att driva sina idéer (Sörbom, 2018). I dessa fall handlar det alltså om att producera och kommunicera en kunskap som utmanar rådande politik och dess kunskapsbas. I den här artikeln sympatiserar vi med Sörboms (2018) definition av våra aktörer “som kunskapsproducenter vilka (som de själva ser det) försöker förbättra policyprocesserna”. Dit räknar vi både det tidiga 1900-talets förbudskritiker som nutida tankesmedjor engagerade i alkoholliberaliseringar.

Centralt för vår undersökning är relationen mellan kunskap och politik, något som ofta förstås utifrån en kunskapsöverförings- eller two-communitieslogik, två skilda universum med få beröringspunkter (Brownson, Royer, Ewing, & McBride, 2006; Graham, Logan, Harrison, Straus, Tetroe, Caswell, & Robinson, 2006; Wehrens, 2014). Studier inom Science and Technology Studies (STS) visar dock att det inte går att skilja olika kunskapsarenor åt utan att det snarare handlar om konstruerade gränser (Gieryn, 1995; Wehrens, 2013) och att relationen mellan kunskap och politik måste ses som en samproduktion (Jasanoff, 2004, 2011; Wehrens, 2014). Oavsett tradition kan man konstatera att relationen mellan kunskap och politik väcker många olika sorters frågor rörande vad kunskap är, samt hur den ska överföras till och användas i policyprocesser.

Många studier fokuserar just på överföringen och användningen av kunskap från forskarsamhället till policyutformningen (Innvaer, Vist, Trommald & Oxman, 2002; Lavis, Oxman, Moynihan & Paulsen, 2008; Mitton, Adair, Mckenzie, Patten & Perry, 2007; Oliver, Innvaer, Lorenc, Woodman, & Thomas, 2014; Wehrens, 2014). När det gäller utomstatliga kunskapsproducenter finns en del forskning, bland annat om att universitetsproducerad forskning utmanas av tankesmedjor och liknande kunskapsproducenters tilltagande betydelse i politiska domäner (Denham & Garnett, 1996; Weaver, 1989). Tankesmedjor och liknande aktörer används som experter i media samtidigt som de själva söker medieljuset för att legitimeras som just experter (Arnoldi, 2007; Osborne, 2004; Rich, 2001). Experter från tankesmedjor kan till viss grad även föredras framför akademiska experter (Steele, 1995), något Arnoldi (2007) menar beror på att de har en högre sammansättning kapital, exempelvis genom sina mediala och politiska kontakter, jämfört med andra universitetsanknutna experter som oftare hyser skepsis mot sådana.

Samtidigt som utomstatliga kunskapsproducenter ofta definieras utifrån sin kunskapsproduktion och påverkansarbete går det inte att frånse deras relation till andra externa aktörer. Sedan tidigare är tobaksindustrins försök att påverka både forskning och policy välkänd. När det gäller alkoholindustrin är detta mindre dokumenterat men de studier som finns visar enligt Hawkins and McCambridge (2014) att alkoholindustrin agerar i alkoholpolicyprocesser genom att snedvrida vetenskaplig evidens, sponsra fristående forskningsorganisationer, ge finansiering till universitetsbaserade forskare, publicera och/eller finansiera vetenskapliga rapporter och tidskrifter eller försöka påverka den allmänna opinionen (Babor, 2009; McCambridge, Hawkins, & Holden, 2013; Stenius & Babor, 2010). Ett exempel är tankesmedjan Demos som har använts av alkoholindustrin för att forma kunskapen i policydebatter genom egna rapporter (Hawkins & McCambridge, 2014).

Svenska studier om tankesmedjor finns i begränsad utsträckning (Allern & Pollack, 2016; Garsten, 2013; Garsten, Rothstein, & Svallfors, 2015; Garsten & Sörbom, 2015; Svallfors 2016; Tyllström, 2013). Tankesmedjan som fenomen är relativt understuderad utifrån frågan om vad de faktiskt gör och hur de blir viktiga i och får in sina idéer i den politiska sfären (Sörbom, 2018). Sörbom anlägger ett organisatoriskt perspektiv på aktiviteterna *nätverkande* och *agendasetting* för att teoretiskt begreppsliggöra och visa att och hur tankesmedjorna skapar förutsättningar för att kommunicera sina idéer in i politiken. Vi vill undersöka en annan aspekt av denna kommunikation, nämligen hur utomstatliga kunskapsaktörer legitimerar sina argument samt med vilken kunskapsunderbyggnad detta sker.

## Material och metod

I det första nedslaget har vi tittat på sex skrifter engagerade i förbudsmotståndet publicerade |åren 1916–1922. Två av dem är författade av företrädare för det förbudskritiska *Förbundet för medborgarfrihet* (Haralds, 1917; Sandberg, 1917) och en är utgiven av *Socialdemokratiska agitationskommittén* ([SAK], 1916) som stod detta förbund (grundat av August Palm) nära. En skrift är utgiven av den mer nykterhetsvänliga men förbudskritiska *Landsföreningen för folknykterhet utan förbud* (Santesson, 1922) och de sista två skrifterna är författade av socialdemokraten och juridikprofessorn Vilhelm Lundstedt (1920) respektive läroverksadjunkten Gideon Forssell (1921). Snarare än en undersökning av representativt material från nej-sidan har avsikten varit att fånga upp resonerande argument bortom den mer endimensionella propagandan som producerades parallellt med dessa, exempelvis av den mer populistiska NEJ-centralen.

I det andra nedslaget har vi studerat Sveriges största tankesmedja – Timbros – alkoholpolitiska engagemang. Timbro grundades 1978 och finansieras av Svenskt Näringsliv (tidigare Svenska Arbetsgivareföreningen). Timbros produktion utgår från “rätten att utforma sitt eget liv, att valfrihet är viktigare än ekonomisk jämlikhet och att politikens makt över människor och företag behöver minska” (timbro.se). Timbro ger ut rapporter, “briefing papers” och böcker, men producerar också krönikor, debattartiklar, reportage och liknande relaterade till en rad politiska frågor. En återkommande fråga är Sveriges alkoholpolitik.

Materialet samlades in genom Timbros hemsida och består av Timbros digitalt publicerade texter mellan åren 2012–2020. För att nå helheten av Timbros alkoholpolitiska engagemang har vi använt oss av breda inklusionskriterier där samtliga 59 texter som innehöll ordet alkohol* inkluderades initialt. 16 texter valdes bort på grund av litet eller inget fokus på alkoholpolitik. Den slutgiltiga empirin består av 43 texter från åren 2012–2020, men endast en text finns från 2012, resterande är skrivna åren 2016–2020.

Kodningsprocessen för de båda nedslagen utgick från en öppen strategi (Saldaña, 2015) kombinerat med ett övergripande fokus på vilken typ av kunskapsanspråk som gjordes samt hur och vilka resonemang som fördes fram med hjälp av dessa. Dominerande empiriska koder var exempelvis folkhälsa, frihet och demokrati medan de teoretiska koderna utgjordes av appropriering, spegling samt att rikta och stjäla rampljuset. Nedan presenteras vår undersökning under tre teman: De alkoholbejakande huvudargumenten, Understöd och Den större samhällsfrågan.

## Friheten och farorna

Under tidigt 1900-tal stod alkoholfrågan högt på den politiska agendan, både i Sverige och övriga västvärlden där flera länder på kort tid även hade infört olika alkoholförbud. Det tidiga 1900-talets alkoholbejakande argumentation kan betraktas som framprovocerad av hotet om en kraftigt restriktiv alkoholpolitik, möjligen också ett alkoholförbud. Denna kontext saknas i början av 2000-talet där vi i några årtionden istället sett betydande alkoholpolitiska avregleringar, exempelvis fler restauranger med serveringstillstånd, förlängda öppettider och utökade möjligheter till privatimport av alkohol.

### De alkoholbejakande huvudargumenten

De huvudsakliga argumenten mot ett alkoholförbud i början av 1900-talet formulerades dels som en förskjutning av problemets orsak, från alkoholen till externa förhållanden, samt genom att beskriva problemet som relativt litet och alkoholkonsumtionen som en frihetsfråga och en privatsak, medan förbudet utmålades som en potentiell fara.

Socialdemokraternas inställning till nykterhetsfrågan och nykterhetsrörelsen var komplicerad och Per Albin Hansson (1930) har i en artikel från 1930 beskrivit det som att alkoholfrågan stod i centrum för en omvälvande maktkamp inom partiet under tidigt 1900-tal. Bortom fraktionsstriden syns konkurrerande perspektiv som endast med svårighet kunde samsas inom ett och samma parti: dels nykterhetsrörelsens föreställning om hur alkoholkonsumtionen bidrog till socialt elände, dels arbetarrörelsens föreställning om hur socialt elände ledde till alkoholmissbruk. Det senare perspektivet, ett slags tidig symtomteori, var också utgångspunkten för Socialdemokratiska agitationskommittén (1916, s. 4) som motsatte sig ett alkoholförbud med argumentet att “den verkliga folknykterheten, […] skapas *endast genom verklig upplysning, bättre bostäder, goda arbetsvillkor och tillgång på underhållande men på samma gång nyttiga förströelser*”.

Agitationskommittén (1916, s. 6) var visserligen noga med att man motsatte sig alkoholmissbruk men också att “ett litet fåtal människor” med “svag karaktär och för klen ambitionskänsla” inte skulle få diktera villkoren för den stora massan “oförvitliga och skötsamma arbetare och bönder”. Istället argumenterade man för “rätten att utan förbud och tvång bestämma över Eder själva i frågan om vad Ni vilja äta och dricka!” (SAK, 1916, s. 7) Kommittén ansåg att “arbetarepartiets medlemmar […] även framdeles [skulle] ha kvar sin rätt att dricka ett glas öl till maten eller i att ett kamratligt lag efter arbetets slut taga sig ett glas punsch eller konjak till kaffet utan att därigenom bliva ansedda som mindrevärdiga medborgare” (SAK, 1916, s. 3). Ett alkoholförbud var helt enkelt i strid med “grundläggande socialdemokratiska frihetsprinciper” (SAK, 1916, s. 3). Nykterhetsrörelsens försök att påverka socialdemokratin fördömdes; deras “världsfrämmande och glädjedödande principer stå i direkt strid med den socialdemokratiska livsfilosofien” (SAK, 1916, s. 4; liknande argument hos Sandberg, 1917 och Forssell, 1921).

Socialdemokratiska agitationskommitténs målande beskrivningar av hur ett alkoholförbud hotade arbetarnas rätt att koppla av med ett glas på sin fritid upphöjdes också till en fråga om var gränsen för politiken skulle dras. Alkoholkonsumtion var en privatsak och på samma sätt som Socialdemokraterna inte la sig i medlemmarnas religiösa tro borde man avstå att “bestämma över om vi taga ett glas dricka till maten, som har några procent högre alkoholhalt än svagdrickat” (SAK, 1916, s. 5). August Palm, grundare av *Förbundet för medborgarfrihet*, menade också att alkoholförtäring närmast var att betrakta som en “dietfråga” och därför knappast något som staten skulle lägga sig i (Johansson, 1997, s. 139). I *Förbundet för medborgarfrihets* skrift författad av Hjalmar Haralds (1917, s. 7) gjorde man på liknande vis en poäng av att nykterheten visserligen föll “inom den offentliga rättssfären, om frånvaron af densamma kränker andras rätt, ordning, offentlig sedlighet eller anständighet”, men också att den inte gjorde det. Nyckelordet här var “andras”; huruvida alkoholkonsumenten själv drabbades eller inte var en privat fråga och därför inget staten skulle lägga sig i (liknande argument i Sandberg, 1917 och Lundstedt, 1920).

Denna systematiska trivialisering av alkoholproblemet är även kärnan i den alkoholbejakande argumentationen i början av 2000-talet då Timbroskribenterna menar att Sverige under de senaste decennierna successivt har avreglerat alkoholmarknaden utan att det har fått de negativa konsekvenser som förutspåddes. En återkommande diskussion utgår från att farhågorna för att ökad alkoholtillgänglighet skulle leda till mer skador, våld och sjukdomar saknar empirisk grund. Dels argumenteras för att den restriktiva politiken är verkningslös, att det inte “finns något samband mellan restriktiv lagstiftning på alkoholområdet och lägre alkoholkonsumtion per capita” (T22, liknande resonemang förs bland annat i T1; T4; T7; T8; T12; T14; T15; T39; T42; T43). Dels argumenteras för att avregleringar inte har ökat skadeverkningarna (T1; T7) utan till viss del även minskat dessa (T1).

Beskrivningen av den svenska alkoholpolitiken som verkningslös eller till och med kontraproduktiv är kärnan i Timbros argumentation. Detta kombineras sedan med ett argument som känns igen från tidigt 1900-tal, om hur det är hur det är roligare, trevligare och innebär mer frihet med en öppen marknad. Att som i den svenska alkoholpolitiken utgå från den så kallade totalkonsumtionsmodellens teoretiska antaganden är att i onödan fokusera på alkoholens negativa konsekvenser och låta “sjukdom, våld och död” (T1) styra alkoholpolitiken.^3^ De farhågor som istället framhävs av Timbro är synen på Sverige som en paternalistisk och glädjedödande stat, vilket tydligt känns igen från det tidiga 1900-talets argumentation.

Men alkoholrestriktioner är inte bara glädjedödande, de kan också vara direkt farliga. För 100 år sedan befarades att ett förbud skulle leda till illegala alkoholhantering av mer hälsovådliga produkter, av “för hela organismen alldeles ödeläggande surrogat” (SAK, 1916, s. 6) som var “långt farligare” (Forssell, 1921, s. 21) än den lagliga alkoholen (Forssell, 1921, s. 21). Det var drycker “av sämsta beskaffenhet och vida mer hälsofarliga än de man förut varit van vid”, mixturer som kunde leda till både blindhet och döden eller till att man ersatte alkohol med narkotika (Forssell, 1921, s. 50; liknande i: Sandberg, 1917, s. 13).

Alkoholförbud var alltså farligt för individen men också för samhället i stort då det kunde leda till sviktande rättskänsla och organiserad kriminalitet. Förbud mot oskyldiga livsstilsval öppnade upp för andra godtyckliga förbud, exempelvis mot kaffe och tobak (Forssell, 1921), och sådana förbud riskerade att förstöra rättskänslan (SAK, 1916). Detta gällde inte endast ett stundande alkoholförbud utan även, enligt Haralds (1917), det redan implementerade Brattsystemet. I grunden handlade det om att alkoholkonsumtion var en vana som var här för att stanna. “Vad tjänar det för övrigt till att utfärda förbud för en vara, som aldrig kan utrotas!”, som Sandberg (1917, s. 12) retoriskt frågade sig. Rimligtvis ingenting, men det var också farligt då “[d]en förbjudna frukten […] alltid [har] sin särskilda lockelse, och den eggas än ytterligare av begäret att motsätta sig lagar, som äro kränkande för känslan av människovärde” (Sandberg, 1917, s. 12).

Tankarna på ett alkoholförbud sågs som en prövning av den unga demokratin och motståndet formuleras som en strävan efter tolerans, att även en potentiell majoritet ska visa fördragsamhet med en eventuell minoritets levnadsmönster. Alternativet var ett majoritetsförtryck med synnerligen farliga verkningar. Att med ett alkoholförbud av planerat slag “kränka någons rätt” ledde till ett “allas krig mot alla”, som Haralds (1917, s. 32) formulerade det. Med förbudet följde husrannsakningar och angiveriverksamhet, kort sagt tyranni, vilket alltid måste bekämpas vare sig det handlade om “fåtals- eller flertalstyranni” (Forssell, 1921, s. 55). Lundstedt (1920, s. 87) beskrev det i liknande termer, som en oproportionerlig kriminalisering som därför var att likna vid ett “*rent terroristiskt skräckregemente*”. I slutänden, menade Forssell (1921, s. 53), följde “utbrottet av ännu ett inbördeskrig, där motståndarna komma att möta varandra med det hat som brukar utmärka inbördeskrig framför andra krig”.

Även om krigsterminologin har tonats ned avsevärt upprepas argumenten i början av 2000-talet då Sveriges alkoholpolitik beskrivs som vilseledande och farlig. Aktörer som Polisen och strukturer kodifierade i form av alkoholskatt, serveringstillstånd och den restriktionslegitimerande totalkonsumtionsmodellen görs skyldiga för tillståndet (T23; T12). Allra farligast är Systembolaget som nyttjar sin folkhälsoretorik som ett “fikonlöv” för en farlig maktutövning:

Som vi har sett har sådana åtgärder föga effekt på hälsan, men de ska inte avfärdas som ofarliga. Förbud och restriktioner kostar pengar och skapar incitament för smuggling och svarta marknader. Straffskatter faller hårdast på de fattigaste. Framför allt reduceras de medborgare som i en demokrati är statens uppdragsgivare och chefer, till mindre vetande undersåtar av politiker och byråkrater. Paternalism är helt enkelt uttryck för en osund makthunger, och Europas stater behöver banta (T9, liknande resonemang förs bland annat i T42; T17).

Det är tydligt att det näringslivsfinansierade Timbro inte vill framstå som ett särintresse. Visserligen driver man en linje som gynnar det alkoholrelaterade näringslivet, alltifrån alkoholproducenter till restauranger, men argumentationen mot regleringar bottnar i ett värnande om folkhälsan och fattiga medborgare. Härigenom kan anklagelsen om statlig paternalism kläs i termer av klassmedvetenhet, en hållning som liknar Socialdemokratiska agitationskommitténs argumentation hundra år tidigare.

I Timbros material synliggörs vad alkoholfrågan har kommit att bli för en fråga. Demokratin, som tidigare används som för att legitimera alkoholbejakande utspel, finns kvar, men kombineras nu i stor utsträckning med alkoholen som en folkhälsofråga. Här är Timbro kluvet. Å ena sidan beskrivs statens hänvisning till folkhälsan som vilseledande propaganda i syfte att värna Systembolaget och de politiker som har gått på denna propaganda ses som svikare av liberala ideal (se till exempel T16). Med folkhälsan som retorisk resurs kan såväl som Systembolaget som rökförbud och sjöfyllerilagen försvaras, men enligt Timbro är detta inte institutioner tillkomna för “folkhälsans skull, utan på grund av en politisk vilja att tvinga andra att leva efter ens egna önskemål” (T37).

Samtidigt vilar Timbros övergripande argumentation på premissen att liberaliseringar av alkoholpolitiken har skett utan att folkhälsoproblemen för den skull ökat och ibland till och med minskat gällande exempelvis den totala alkoholkonsumtionen, den alkoholrelaterade brottsligheten och det alkoholrelaterade dödliga våldet T12; T2; T4). Den genomgående tendensen att skriva fram frånvaron av negativa konsekvenser som skador, våld och andra folkhälsorelaterade aspekter erkänner samtidigt bekämpande av dessa som legitima alkoholpolitiska mål. Dessutom presenteras också nya folkhälsoaspekter, som att [f]rihet är bra för hälsan” (T1).

### Understöd

Ovan redovisade alkoholbejakande argument har legitimerats med hänvisning till såväl historiska exempel och andra bittra erfarenheter som utsagor hämtade från media och forskningen. Argumentationen mot det tidiga 1900-talets alkoholförbud byggde i stora delar på att det helt enkelt inte fungerade (Forssell, 1921; Lundstedt, 1920; SAK, 1916). Här var det historiska exemplet en resurs och med hänvisning till romerska förbudet mot kristendom eller det kinesiska opiumförbudet i slutet av 1700-talet och början av 1800-talet, visade Lundstedt (1920) att vissa saker helt enkelt inte gick att avskaffa medelst lagstiftning. Även Sandberg (1917, s. 7) hämtade avskräckande exempel ur ett förflutet som bjudit på mängder av förbud för att visa hur dessa ledde till “raka motsatsen av vad man beräknat”:

Spriten har varit förbjuden, kaffepannan utan nåd förbruten, husrannsakan var tillåten, myndigheternas sax verksam för att avklippa sådant kvinnobjäfs, som var stridande mot förordningarna, på rökaren och snusaren skulle näsborrar och mungipor uppskäras. Men vad hjälpte allt detta. Förordningarna stannade i de flesta fall på papperet eller föranledde trakasserier.

Förbud var inte bara verkningslöst utan bidrog dessutom till ökad kriminalitet. Om vi undantar spritförsäljning, som ju efter ett infört förbud blir illegalt just på grund av förbudet, så handlade det om lönnbränning och smuggling (Forssell, 1921; SAK, 1916). Här kunde andra länders spritförbud tjäna som exempel, som Finland där “förbudet som genom att fresta med ekonomisk vinst sänkt den svenskfinska skärgårdsbefolkningen i laglöshet och dryckenskap” (Forssell, 1921, s. 59). Därtill befarades, med exempel hämtade från de amerikanska erfarenheterna, en omfattande korruption bli resultatet av ett alkoholförbud. Det handlade dels om direkt lagvidrig verksamhet där poliser och politiker köptes för att se mellan fingrarna med illegal verksamhet, men också om den mer subtilt korrumperande verkan som kom sig av en karriäristisk anpasslighet till nykterhetsfrågan när politiker sökte säkra sina positioner (Forssell, 1921; SAK, 1916). Med Sandberg (1917, s. 8) hade nykterheten “blivit en [*sic.*] språngbräde för politiska ‘Streber’: ett de andefattiga yrkespolitikernas medel för att uppnå politisk makt”. Den tydliga överrepresentationen av förbudsvänner i riksdagen var en konsekvens av detta (Santesson, 1922). Men, som visats ovan, kunde det gå ännu värre och leda till ren terror eller, med Forsells (1921, s. 32) exempel hämtade från förbudslandet USA, ett slags “revolverpolitik”:

Förbudspolisen skonar varken liv eller egendom. Tågen hejdas mitt i natten och polismännen tränga in i sovvagnarna, även kvinnornas och barnens, bryta upp koffertarna, genomsöka kupéerna med sina blindlyktor och uppmana passagerarna, med revolvern för bröstet, att utlämna möjligen medhavda spritdrycker. Vid minst motstånd riskeras att bli nedskjuten, vilket nyligen hände två män som protesterade mot förbudstjänstemännens hänsynslösa uppträdande.

Det avskräckande exemplet syftar rimligtvis mot en faktabaserad argumentation för den egna ståndpunkten. Samtidigt var Forssell (1921, s. 10 f) medveten om att känslan spelade lika stor roll som förnuftet i denna fråga, att den egna hållningen grundades i “våra egna vanor, vår smak, våra tycken och sympatier”, att inställningen till ett alkoholförbud var “psykologiskt, icke blott logiskt motiverad”. Men detta hindrade inte Forssell från att själv empiriskt söka underbygga sin bild av vad ett alkoholförbud skulle leda till. Exempel hämtades frekvent från länder som redan hade infört alkoholförbud, vanligtvis via beskrivningar i dagspressen. Så kunde exempelvis Forssell, med hänvisning till ryska förhållanden, varna för att alkoholförbud ledde till ökat narkotikabruk. Och med Norge och Finland som exempel gjorde Forssell (1921) troligt att förbjuden olaglig sprit endast skulle ersättas av olaglig sprit, något som enligt Haralds (1917), utifrån en notis i Hufvudstadsbladet, även gällde i Ryssland. Och den revolverpolitik, det skräckregemente, som befarades utifrån amerikanska exempel, kunde även hävdas med hänvisning till Finland. Det visste Forssell (1921) med stöd i Aftonbladet som i sin tur citerade en promemoria i förbudsfrågan ställd till den finska överståthållaren.

Tidningsreportage var en vanlig kunskapsbas men vid några tillfällen refererades vetenskapliga auktoriteter, som då Haralds (1917, s. 4) redovisade medicine professor Ulrik Quensels ifrågasättande av alkoholens “absoluta skadlighet”. En tredje variant återfinns hos Lundstedt som inte så mycket refererade vetenskapliga auktoriteter som att han intog denna roll själv. Som professor i juridik hänvisade Lundstedt flitigt till egna arbeten där principerna för hans motstånd mot alkoholförbud gick att spåra. Det handlar företrädesvis om ett slags rättsfilosofisk härledning där det utifrån vissa postulat går att resonera sig fram till slutsatserna, inte om några empiriskt understödda scenarier.

Forssells flitiga bruk av dagspressen, Haralds hänvisningar till noga utvald medicinsk sakkunskap och Lundstedts rättsfilosofiska självrefererande väcker frågan om vad som var legitim kunskap om alkoholförbudets verkningar. Det går nog inte att besvara den frågan på annat sätt än att legitim kunskap var den kunskap som understödde den ståndpunkt som man redan hade intagit. De undersökta skrifterna är överlag väldigt empirisvaga och ingen av dem kan göra anspråk på att vara en undersökning i vetenskaplig mening. Kunskaper om sakförhållanden understödde undantagslöst den egna ståndpunkten och nästan inga försök görs att ens identifiera konkurrerande utsagor, om än endast för att vederlägga dem. Ett enskilt undantag återfinns hos Haralds, vars skrift har till yttersta syfte att desavouera Kontrollstyrelsen, men då handlar det inte så mycket om att falsifiera påstådda sakförhållanden som att misstänkliggöra kunskapsproducenten, Kontrollstyrelsens aktuarie Einar J:son-Thulin. Denne saknade enligt Haralds helt kvalifikationer att utreda nykterhetsfråga, något som kom sig av att han var statsvetare. “Medicinska, juridiska (inklusive nationalekonomiska) och naturvetenskapliga fackmeriter saknades”, skriver Haralds (1917, s. 13), och ger därmed en antydan om vilka discipliner som hade rätt sorts kunskap för att utreda frågan.

Den största skillnaden mellan det tidiga 1900-talet och det tidiga 2000-talet ligger inte i valet av argumentationslinjer utan snarare i hur dessa förstärks för den senare perioden. I materialet från åren 1916–1922 legitimerades resonemangen utifrån utblickar i tid och rum, med historiska, internationella och mediala referenser, samt i något fall med den egna vetenskapliga auktoriteten. Även Timbro nyttjar media, historiska och internationella jämförelser frekvent i sin argumentation, exempelvis genom att sammanblanda expertis och journalistik i texter som refererar till debattartiklar, politikers utspel eller mediala uttalanden från forskare. I sådana exempel härrör resonemangen och uttalandena inte nödvändigtvis från faktisk forskning men samtidigt tillskrivs de en stor tyngd. Journalistiska texter jämställs även med vetenskapliga texter, till exempel för att argumentera för att Sverige har för starkt alkoholmonopol, ifrågasätta Systembolaget, motsäga siffror om smuggling eller när en akademisk artikel och en radiodokumentär tillsammans benämns som “flera kartläggningar” som vittnar om en “utbredd mygelkultur” (T42, se även t.ex. T4; T8)

När det gäller vetenskaplig auktoritet skiljer sig Timbro dock från det tidiga 1900-talets aktörer i att de oftare refererar till etablerad och statligt finansierad forskning. Argumentationen i Timbros huvudfråga – ökade liberaliseringar – bygger genomgående bygger på offentlig expertis och kunskapsproduktion, exempelvis från organ som Stockholm förebygger alkohol- och drogproblem (STAD), Centralförbundet för alkohol- och narkotikaupplysning (CAN), Brottsförebyggande rådet (BRÅ), Folkhälsomyndigheten (FoHM), Centrum för socialvetenskaplig alkohol- och drogforskning (SoRAD) eller Institutet för social forskning (SOFI). Det här är kunskapsproducenter som ofta nyttjas i argumentationen för en mer restriktiv alkoholpolitik, men Timbro nyttjar dem istället för att ifrågasätta den svenska alkoholpolitikens interventioner, förklaringsmodeller och restriktioner i allmänhet och Systembolagets monopol i synnerhet. Rådande alkoholpolitik beskrivs här ofta som en ovetenskaplig, oseriös och godtycklig sköld för Systembolagets existensberättigande. Alkoholpolitikens teoretiska alibi, totalkonsumtionsmodellen, utmålas som ovetenskaplig och sammanfattningsvis beskrivs staten som paternalistisk, oseriös och vilseledande (T3; T4; T6; T8; T11; T15; T16; T20; T21; T24; T25; T34; T42). Ett exempel är Systembolagets beskrivning av att alkoholmonopolet räddar 2000 liv per år, byggt på en studie från Stockholms universitet (Norström, Miller, Holder, Österberg, Ramstedt, Rossow & Stockwell, 2010.) som redovisar en samvariation men inte nödvändigtvis ett orsakssamband. Som Timbro konstaterar hade den “siffran […] lika gärna kunnat vara tagen ur luften” (T3).

Även om Timbro avfärdar “sjukdom, våld och död” (TI) som en rimlig utgångspunkt för svensk alkoholpolitik så återvänder de själva ofta till dessa katastrofbeskrivningar för att kritisera totalkonsumtionsmodellen och Systembolagets problematiska verkningslöshet. Med andra ord så bekräftar de relevansen av skade- och våldsargumentationen genom att upprepa och prioritera den i sina texter. Argumentet att Systembolaget är grundläggande viktigt för folkhälsan utmanas återkommande, exempelvis med hänvisning till alkoholforskarna Ramstedt (2010) och Trolldal och Leifmans (2015) forskning om att alkoholskador inte har ökat efter avregleringarna. Att peka på studiers påstådda orsakssamband för att betona att det i själva verket handlar om samvariationer, samtidigt som samvariationen mellan ökade liberaliseringar och minskade negativa konsekvenser såsom alkoholrelaterat våld och skador betraktas som ett möjligt orsakssamband, är två motstridiga processer som sammantaget utmanar rådande alkoholpolitik och dess interventioner.

Timbros argumentatoriska strategi skiljer sig ordentligt från hur förbudsmotståndarna hanterade förbudsivrarnas produktion ett sekel tidigare. Den kunskapsproduktion i form av forskning och utredningar som syftade till att stärka ett förbud ignorerades då i stort sett i det undersökta materialet. Timbro arbetar i stället med en återkommande formel som både manifesterar och utmanar den vetenskapliga expertisen. Erkända auktoriteter inom alkoholforskningen presenteras just som sådana samtidigt som deras vetenskapliga anspråk undermineras då man visar hur fel de hade om exempelvis alkoholliberaliseringarnas negativa konsekvenser (såsom ökad alkoholrelaterad dödlighet, våld eller skador), eller då annan forskning tas i bruk för att visa på andra orsakssamband. Exempelvis hänvisas till nationalekonomen David Sundéns dom över Systembolaget som ett verkningslöst monopol, ett kunskapsanspråk som stärks av att Sundén hänvisar till sina egna skrivningar om detta i en rapport från Expertgruppen för studier i offentlig ekonomi (ESO), en kommitté knuten till Finansdepartementet (T39). Det ger onekligen tyngd åt argumentationen att på detta sätt hänvisa både till en etablerad forskare och staten, i form av Finansdepartementet, och ESO-rapporten blir snabbt en ny referens för kommande texter att förhålla sig till, exempelvis för att stärka argumenten om att Systembolagets monopol inte fyller någon funktion och inte heller förbättrar folkhälsan (se till exempel T42).

Mer väntat men kanske också mindre verkningsfullt är att Timbro nyttjar näringslivsdrivna organ som Handelns utredningsinstitut (HUI) i sin kritik av alkoholpolitiken, liksom den återkommande presentationen av det ideologiskt tendensiösa Nanny State Index (T22; T10; T31). Bakom Nanny State Index står författaren och journalisten Christopher Snowdon som också är knuten till högertankesmedjan Institute of Economic Affairs (IEA) och precis som namnet indikerar så redovisas i Nanny State Index olika länders grad av förmynderi och paternalistisk politik. Om de traditionella experterna således används för att argumentera för att ökad liberalisering inte har ökat de negativa konsekvenserna, så argumenteras med stöd i Nanny State Index för att restriktioner inte ökar förutsättningarna för en bättre folkhälsa, för att utmåla EU-ländernas och framför allt Sveriges folkhälsopolitik som paternalistisk och “drakonisk” (T30) eller som “de små stegens tyranni” (T31).

### Den större samhällsfrågan

Alkoholen har alltid varit en katalysator för andra frågor, ofta breda samhällsfrågor i tiden: industrialiseringen, krigsmaktens status, kvinnors politiska rättigheter, urbanisering och modernisering, etc. (Edman, 2015; 2016). Alkoholfrågan tog för hundra år sedan form i ett klimat av kvardröjande nationalism och nationalromantik där “[a]lla klarsynta fosterlandsvänner måste ena sig i kamp mot den hotande olyckan av ett förbud”, som Forssell (1921, s. 62) formulerade det.

Den stora samhällsfrågan dessa år var dock den spirande demokratin. Även om flera steg återstod mot en mer fullkomlig demokrati går det att räkna den allmänna rösträttens dagar från det första val som kvinnor fick rösta i 1921. 1922 års förbudsomröstning skulle därför bli det andra val som myndiga svenskar av bägge könen fick delta i och av rädsla för att de mer nykterhetsvänliga kvinnorna skulle bidra till förbudets införande beslutades att valsedlarna skulle könsmärkas, underförstått att ett av kvinnor framröstat alkoholförbud skulle kunna bestridas (Bengtsson & kvinnor, 2011). Därtill hade Socialdemokraterna beslutat att inte bidra till förbudets genomförande med mindre än att två tredjedelar röstade för dess införande (Johansson, 1997). I strikt demokratiska termer var det knappast en förutsättningslös omröstning.

Men dessa säkerhetsåtgärder räckte inte för vissa debattörer som snarare ville utdöma en ja-seger som odemokratisk och därmed illegitim redan på förhand. En retorisk manöver som tjänade detta syfte var att göra antaganden om valda delar av befolkningen och dess åsikter. Oavsett folkviljan så var ett förbud något som “*icke* omfattas av den stora massan av landets tänkande arbetare”, som Socialdemokratiska agitationskommittén (1916, s. 2) uttryckte det, där det underförstådda budskapet var att just arbetares åsikter om detta borde tillmätas ett större värde och att om de till äventyrs ändå var förbudsvänliga så var de inte tänkande. Agitationskommittén (1916, s. 5) sökte i första hand påverka Socialdemokraterna att följa dess linje och återkom därför till argumentet att partiet borde ändra sitt program “så att det står i verklig överensstämmelse med arbetareflertalets mening och ställning till de alkoholhaltiga dryckerna”.

Liknande argument framfördes av Lundstedt (1920, s. 37) som gärna såg att man stukade landsbygdens nykterhetssträvanden genom att ta särskild hänsyn till folkviljan i “större städer och industricentra på landet”. Men hotet kom särskilt från kvinnorna vilka därför borde tillmätas mindre vikt i den kommande omröstningen (eller hur man nu ska förstå det snart realiserade kravet på att skilja deras röster från männens): “Hur osympatiskt det än må uppfattas av kvinnorna just nu, när de erhållit sin så varmt eftersträvade rösträtt, förefaller det mig såsom en alldeles extra oförnuftighet, att i en sådan fråga som denna icke göra skillnad mellan män och kvinnor” (Lundstedt, 1920, s. 37 f.).

Om agitationskommittén och socialdemokraten Lundstedt främst argumenterade strategiskt för att påverka Socialdemokraterna så bistod Forssell med ett principiellt mer sofistikerat demokratiargument, ett slags minoritetsskydd som inte sällan ingår i definitionen av en välfungerande demokrati (Petrén, 1988). Forssell (1921, s. 15) varnade för “de avarter av demokrati som tyckas tro att majoriteten under alla förhållanden skall ha rätt att påtvinga minoriteten sin vilja”. Forssell (1921, s. 15) skrift publicerades 1921, samma år som den allmänna rösträtten tillämpades för första gången, och han spådde att ett sådant majoritetsförtryck kunde leda till att “både frihet och demokrati grävt sina egna gravar”. Människor värderade “den personliga friheten [högre] än den politiska” och om demokratin brukades för att “förstöra trevnaden i samhället, då kommer även demokratiens dagar snart att vara räknade” (Forssell, 1921, s. 17). Forssells oro för den unga demokratins bruk av majoritetsprincipen delades av Lundstedt (1920, s. 88) som varnade för att “[r]ättsliga monstra” skulle bli “demokratiens första frukter”. Lundstedt underströk att demokratin endast var en form för beslutsfattande som, om än sympatisk, precis som despotin kunde resultera i både goda och onda beslut. Här stod större värden på spel och ett “bedömande av demokratiens slutliga värde för ett folk” kom slutligen av på “om den inriktats i det godas eller ondas tjänst” (Lundstedt, 1920, s. 89).

Enligt Forssell (1921, s. 18) borde ingen lag “i fråga om mat och dryck och kläder” få stiftas utan en majoritet på minst 90 procent och han var därför kritisk till att nykterhetskommitténs föreställning om en erforderlig “stark folkvilja” betydde 60 procent. Forssells kommunicerande argument vantrivsel och demokrati bar redan i sina utgångspunkter på en utilitaristisk princip som utan svårigheter landade i slutsatser med viss kvantitativ precision. Den bästa lösningen, “‘det allmänna bästa’”, översattes av Forssell (1921, s. 20) till “‘den största möjliga lycka åt det största möjliga antal människor’”. Detta skulle kunna tolkas som att ett förbud inte kunde försvaras om en majoritet bara blev lite lyckligare av ett förbud samtidigt som en minoritet mycket olyckligare av detsamma. Folket är en stark retorisk resurs men bruket av detta argument är något godtyckligt. Stora delar av folket måste räknas bort om det rätta folket ska kunna stärka argumentationen. Könstillhörighet, geografisk position (landsbygd/stad) och föreställningar om vem som är en tänkande arbetare är exempel på sådana avgränsningar.

Hundra år senare ligger fokus fortfarande på folket, om än i dagens övergripande tankefigur folkhälsan snarare än folkviljan. Statens folkhälsoarbete beskrivs som en falsk legitimering av alkoholpolitiken; den så kallade folkhälsolobbyn är enligt Nanny State Index-grundaren Snowdon en “statssponsrad verksamhet, i praktiken förgreningar av staten som lobbade för att regeringen skulle genomföra en politik den redan var hågad att genomföra” (T17; se även: T2; T13; T16; T27; T37). Det är ett ifrågasättande av en illegitim paternalism som formuleras i termer av att vara på medborgarnas sida, som att folkhälsolobbyn är ett “elitprojekt för att städa upp arbetarklassen” (T17) eller i beskrivningar av “en alkoholpolitik utan ett uns förtroende för medborgarnas förmåga att ta ansvar för sitt eget liv” (T5), en “alkoholpolitik som konstant sugit ut arbetarklassen men varit ogenerat generös gentemot de välbärgade och opinionsbildande klasserna” (T42; se även: T10; T12; T18; T29; T30; T31; T32; T37; T43; samt mer i förbifarten: T26; T27; T28; T35; T36; T38; T40; T41). Det klassperspektiv som Socialdemokratiska agitationskommittén stod för när man hundra år tidigare hävdade arbetares rätt att ta en sup till maten har nu axlats av Timbro.

Att tala om utsatta grupper av medborgare eller om deras inskränkta rättigheter är ett effektivt sätt att argumentera för politisk förändring (se Winter, 2019; Winter, 2020). Denna medborgardiskurs återfinns också i beskrivningar av politiken som inte bara paternalistisk utan också godtycklig, ovetenskaplig och inkonsekvent samt i de mer hårdföra antipaternalistiska diskussionerna (se till exempel: T12; T14; T5; T9; T18; T21; T29; T31; T37). Med andra ord går paternalismtemat ofta hand i hand med en argumentation om att folkhälsopolitiken egentligen inte främjar folkhälsan. Det är nykterhetslobbyn snarare än evidensbaserad kunskap som styr politiken; politiker har inte folkets bästa utan ekonomiska motiv framför ögonen, och de suger dessutom ut arbetarklassen med sin politik.

Timbros klassmedvetna solidaritet är något begränsad och används uteslutande för att stärka det antipaternalistiska argumentet. Men det är inte bara arbetarklassen, utan befolkningen i sin helhet, som missgynnas av en alkoholpolitik som enligt Timbro motiveras av omsorg de knappt sex procent av befolkningen som har alkoholproblem (T19). Argumentet, hur en liten andel svaga karaktärer inte ska få förstöra för den stora skötsamma massan, känns igen från förbudsstriden (Forssell, 1921; Haralds, 1917; SAK, 1916).

Kritiken av det Timbro kallar för folkhälsolobbyn är en kritik av den statligt sanktionerade kunskap som legitimerar alkoholpolitiken. Som vi visade ovan kritiseras denna expertis men samtidigt nyttjas den för att bestrida samma politik i en process som kan liknas vid ett slags politisk appropriering. Detta sker dels genom att rikta strålkastarljuset mot och “blotta” kunskapen från de myndigheter som via (folkhälso)politiska mål propagerar för restriktiv alkoholpolitik, genom att så att säga klä av dem, dels genom att i nästa led stjäla rampljuset och återanvända de politiska klädnader som de just har avlägsnat. Ljuset riktas *mot* myndighetsaktörer snarare än *från* dessa för att visa hur fel de har – skador har inte ökat, politiken uppfyller inte folkhälsomålen – varpå nya folkhälsomål och kamper (exempelvis för utsatta medborgare) återuppstår i Timbros förslag. Detta sker genom att mer konkret ge sig in i formulerandet av vad folkhälsa faktiskt är och vem som ansvarar för den. På samma gång som statliga folkhälsoargument undermineras, används alltså ett modifierat folkhälsoargument för att stärka Timbros linje.

Att Timbro så tydligt erkänner folkhälsomål skulle kunna ses som ett uttryck för att folkhälsoargumenten accepteras som rimliga av liberala marknadsdebattörer. Detta skulle kunna legitimera dem som berättigade orsaker till att inskränka friheten förespråkad av Timbro, men framför allt ter sig det ständiga manifesterandet av och kopplingen till folkhälsofrågan som en strategi för Timbros skribenter att göra sig hörda. Ett sätt att bruka den gemensamma folkhälsodiskursen är då att tillföra ny kunskap till den. Genom att peka på statens folkhälsoivrande kunskapsbas som ofullständig bidrar Timbro inte bara till kritik men också till nya samband och lösningar för folkhälsorelaterade problem. Smuggling (som även var ett tema under förbudsstriden) och gränshandel lyfts exempelvis som stora problem för en ökad konsumtion i allmänhet och för Sveriges unga befolknings hälsa i synnerhet. Här går författarna tydligt med på att folkhälsan ska stärkas och skyddas, och idealet att svenskarna ska dricka mindre ligger kvar i lösningen att avskaffa alkoholmonopolet.

Men Timbro bistår också med vissa omformuleringar av folkhälsomålen. Exempelvis kopplas folkhälsan till ett narrativ om individens frihet, trivsel och glädje, “vardagens och helgens små glädjeämnen” (T10). Återigen finns en tydlig relation till det tidigare förbudsstridsmaterialets diskussion om glädjeämnen (Forssell, 1921; SAK, 1916), “‘den största möjliga lycka åt det största möjliga antal människor’” (Forssell, 1921, s. 20), samt sättet att se på politiken som glädjedödare (SAK, 1916. Skillnaden är att glädjen nu också formuleras som en folkhälsoparameter att ta hänsyn till. Genom att poängtera vikten av folkhälsan kan Timbro nu ta över ansvaret för denna fråga och i exempelvis rapporten “Förbudsministern” (T2) utvecklas det individuella självbestämmandet som en “konstaterad folkhälsofaktor” i en diskussion om hur dåvarande folkhälsominister Gabriel Wikström förbereder 15 nya förbud på alkohol- och tobaksområdet:

Wikström gör också gemensam sak med lobbyorganisationer som vill motverka allt bruk av alkohol och tobak, en ideologisk uppfattning som går långt bortom folkhälsoskäl. Inte i en enda artikel, en enda intervju eller ett enda dokument nämner Gabriel Wikström något om värdet av att människor får bestämma själva, ens över dessa vardagslivets nöjen. Detta trots att självbestämmande är en konstaterad folkhälsofaktor, i exempelvis arbetslivet (T2; liknande resonemang i: T9; T10; T37; T31).

Alkoholpolitiken bör sammanfattningsvis utformas i relation till folkhälsopolitiska mål. Förbud och restriktioner är en paternalism i strid med eftersträvad liberalisering men ändå något som skulle kunna motiveras av folkhälsoskäl, eller åtminstone borde, men brister i göra det. En statlig folkhälsopolitisk agenda framställs som relevant, även om det aktuella *innehållet* i den inte accepteras. Timbro argumenterar inte bara mot att ministerns förbud kan motiveras av hälsoskäl, utan också mot att dessa skulle grunda sig i legitim (av experter sanktionerad) kunskap om vad folkhälsorelaterade åtgärder är, eftersom de inte tar hänsyn till folkhälsofaktorn självbestämmandet. Den befintliga alkoholpolitiken separeras på så vis från och sägs “gå långt bortom” folkhälsomålen när den kopplas till specifika lobbyorganisationer som både påverkat och gynnas av Wikströms politik. I liknande resonemang relateras detta också till den farliga makten som ideologiska organisationer eller Systembolaget får genom att använda illegitima folkhälsoargument (T9; liknande resonemang i: T42; T17).

## Avslutning

Vad var alkoholfrågan i början av 1900-talet egentligen för fråga för utomstatliga kunskapsproducenter? Och vad har den kommit att bli? Det tidiga 1900-talets aktörer formulerar sig i en kontext där ett förbud som står och trampar i farstun måste avstyras. Att argumentationen så tydligt fokuserar på att just vara mot förbudet är därför inte konstigt. Trots det enhetliga målet har vi funnit en bred argumentationslinje som på flera fronter angriper idén om ett förbud. Emellanåt framstår alkoholfrågan som en icke-fråga. Det handlar om att förminska problemet, framhäva betydelsen av glädjen kopplad till alkoholen, att göra det till en privatsak, ett livsstilsval som precis som valet av kläder är något som staten självklart ska hålla sig borta från. Men konsekvenserna av ett förbud är därför inte bara beklagliga; de utmålas stundtals som direkt farliga och samhällsomstörtande, något som får alkoholen att också framstå som oerhört viktig.

När det gäller 2000-talet finns inget motsvarande akut stundande förbudshot. Istället är det existerande institutioner, lagar och regler som Timbro ser som både verkningslösa och hotfulla. Trots de kontextuella skillnaderna återfinns flera av de tidigare förbudskritiska resonemangen i de senares pläderingar för en mer liberal alkoholpolitik. Först och främst reflekteras den förra tidsperiodens formulering av problemet som litet i Timbroskribenternas resonemang om att Sverige under de senaste decennierna successivt har avreglerat alkoholmarknaden utan att det har fått de negativa konsekvenser som förutspåddes. Sverige beskrivs som en paternalistisk och glädjedödande stat och den befintliga alkoholpolitiken som verkningslös i relation till dessa påstådda negativa konsekvenser. Värdet av den enskilda individens frihet som större än värdet av en politik där medborgare tvingas foga sig i restriktioner av solidaritet för de som skulle få problem av en mer liberal politik ekar också förbudsstriden. Sveriges alkoholpolitik formuleras under våra bägge undersökningsperioder genomgående som oseriös, vilseledande eller farlig.

Spänningen mellan alkoholfrågan som en fråga om frihet och samtidigt en fråga om fara och av avgörande betydelse för det framtida samhällets utformning genomsyrar också tydligt bägge tidsperioderna. Skillnaden är att de kopplas till två olika övergripande samhällsfrågor. I den förra alkoholpolitiska debatten framhävs demokratin som det stora värde som står på spel medan folkhälsan blir en obligatorisk strävan att förhålla sig till i den senare debatten. Men trots att alkoholfrågan har kommit att alltmer formuleras i termer av folkhälsa har Timbro en ambivalent inställning till den rådande alkoholpolitikens problemformulering. Medan förbudskritikerna i början av 1,900-talet bestrider själva existensen av ett alkoholproblem så erkänner Timbroskribenterna i början av 2,000-talet detta problems realitet som en slags strategi för att sedan bestrida att problemet kan lösas med nuvarande alkoholpolitik.

Argumentationslinjerna är som sagt förhållandevis lika trots att både aktörer och övergripande samhällsfråga skiljer sig åt i de båda fallen. Argumenten understöds med historiska, internationella, mediala och vetenskapliga belägg som visserligen återfinns i olika utsträckning i de båda tidsperioderna, men när det gäller vetenskapligt stöd finns en stor skillnad. Förbudskritikerna i början av förra seklet har inget större behov av att stärka sin argumentation med vetenskaplig förankring. Snarare var folkviljan viktig här. Allmänheten representeras i form av den skötsamme medborgaren som drabbas av ett förbud, folkviljan manifesteras på olika sätt genom majoritetsbeslut eller att den anses vara utsatt för majoritetsförtryck, och folket kan vara arbetare, kvinnor, tänkande respektive otänkande arbetare, något som ger breda förutsättningar för att stärka argumentationslinjerna. Många studier har visat hur traditionell expertis kan utmanas eller kompletteras med annan typ av kunskap från exempelvis allmänheten eller patientgrupper (Collins & Evans, 2002; Carolan, 2006; Winter, 2019; Rabeharisoa & Callon, 2004). Men här är det frågan om att folkviljan – som legitimerande understöd – ersatte snarare än utmanade traditionell expertis.

Timbro nyttjar däremot kunskap från både inom- och utomstatliga kunskapsproducenter för att visa att liberaliseringar på alkoholområdet inte har lett till ökade negativa konsekvenser, att det finns en diskrepans mellan folkhälsomål och expertis samt för att beskriva rådande politik som ideologisk, ovetenskaplig och godtycklig i sitt kunskapsbruk.

Legitimering genom användning av befintlig kunskap – främst inomstatligt producerad forskning och kunskapsunderlag – sker således i betydligt större omfattning i det senare materialet, något vi har diskuterat i termer av att rikta och stjäla rampljuset; ljuset riktas mot myndighetsaktörer snarare än från dessa för att visa hur fel de har. Genom detta fokus möjliggörs två saker: 1) en underminering av befintliga folkhälsomål (skadorna har inte ökat; politiken uppfyller inte folkhälsomålen) och de agendor som ligger bakom dessa, samt; 2. ett omformulerande av vad folkhälsomål och sakfrågor (exempelvis att tala för fattiga medborgare) kan vara. Här vill vi betona hur just *användning* av kunskap bör ses som en produktiv praktik bortom själva *upprepningen* av någons kunskapsanspråk. Vid en första anblick använder snarare än producerar Timbro den befintliga kunskapen från BRÅ, CAN, SoRAD och liknande aktörer. Samtidigt är Timbros anspråk och argument avhängiga dessa befintliga kunskapsanspråk som inte bara legitimerar, utan också utgör en förutsättning för produktionen av argument såväl som agendasättande.

Att agendasättande är viktigt för att påverka vad ett visst policyområde bör satsa på är en självklarhet i studier som rör policyprocesser och utomstatliga kunskapsproducenter såsom tankesmedjor (Rich, 2005; Sörbom, 2018). Medan tidigare studier har visat på hur tankesmedjors samarbeten påverkar andra aktörers agendor – Sörbom visar exempelvis hur ett organisatoriskt perspektiv och *partiell organisering* (Ahrne & Brunsson, 2011) kan användas för att förstå tankesmedjors nätverk – har vi här en motsatt process. Med andra ord, att utomstatliga kunskapsproducenter i form av tankesmedjor – hur mycket i opposition den än befinner sig – ändå utgår från “det som redan sagts” i befintlig alkoholpolitik. Teoretiskt ekar detta en konversationsanalytisk tradition gällande vikten av sekvenser (Sacks, 1995). Att något först sägs om folkhälsopolitik kan förstås som att det sätter agendan för samtalet, eftersom den som kronologiskt kommer in senare i interaktionen måste förhålla sig till vad som först har sagts. Vi menar inte att statens alkoholpolitik därmed uteslutande faktiskt sätter agendan, men likväl att den bildar en utgångspunkt för vilken typ av argument som sedan kan formuleras.

Man kan fråga sig varför Timbro inte tydligare går i opposition mot befintlig politik utan att samtidigt “gå med på” dess ideal och problemformuleringar. Analysen av detta som en fråga om att rikta och stjäla rampljuset visar dock hur den befintliga folkhälsopolitiken blir en produktiv förutsättning för vilken typ av kunskap som kan föras fram. Detta eftersom aktörerna ges möjlighet att omförhandla själva grunden gällande vad folkhälsopolitik kan vara. Detsamma kan vi se i förbudskritikernas användning av demokratin. Det är inte gångbart att ifrågasätta varken demokratin eller folkhälsans betydelse men genom att rikta ljuset mot dessa “stjäl” man så att säga frågan för dagen och kan därigenom laborera med innehållet. Strålkastarljuset blottar och klär av kunskapen, och sedan stjäls ljuset genom att de just avlägsnade politiska klädnaderna återanvänds i ny tappning. Vår tolkning är helt enkelt att det är mer effektivt att låta argumenten bada i folkhälsa (eller i demokrati för den förbudskritiska delen) för att kunna “spela bredare” med en egen folkhälsoretorik. Med andra ord är det frågan om en sorts politisk appropriering. Denna nya folkhälsoretorik och dess förslag kan erbjudas, tilltala och göra sig hörd på fler arenor och av fler aktörer som inte tidigare sympatiserat med liberal alkoholpolitik. Ny publik kan alltså vinnas genom denna hänsyn till kärnfrågan folkhälsa, samtidigt som den faktiska folkhälsopolitiken hålls på tryggt avstånd.

Agendasättande är i högsta grad en flersidig process där utmanande utomstatliga kunskapsanspråk hittar nya sätt att påverka. Den statligt sanktionerade kunskap som legitimerar alkoholpolitiken kritiseras, men nyttjas ändå för att bestrida samma politik. Den liberala argumentationen kring att uppluckra marknaden föds, göds och inspireras således ur den folkhälsopolitik de vill förkasta. Huruvida detta är en medveten strategi respektive hur utbredd den är återstår att studera, men det är tydligt att mekanismerna förekommer frekvent i materialet från Timbro. Det är inte heller sannolikt att Sveriges största tankesmedja så pass ofta av en slump påverkas av statens sätt att formulera sig om folkhälsa. På samma sätt som nätverk mellan tankesmedjor och andra aktörer inte är slumpmässiga utan beslutade praktiker för att få andra aktörer att använda tankesmedjans idéer (Sörbom, 2018), kan man utgå från att tankesmedjor gör väl avvägda och medvetna strategiska val gällande vad som publiceras och varför.

Metaforen rikta och stjäla rampljuset har varit särskilt användbar för att förstå detta. Trots att det är något som spontant kan iakttas i olika utsträckning i de flesta politiska kunskapsprocesser, har tidigare studier ofta fokuserat just på överföring av kunskap till och påverkan på politik. Vi har visat hur befintlig politik och det kunskapsunderlag den bygger på återanvänds av utmanande kunskapsproducenter. Deras kunskapsanspråk görs på grund av, tack vare, och skulle inte kunna göras utan detta återbruk. Utifrån detta bör också relationen mellan kunskap och politik i termer av enkelriktad kunskapsöverföring mellan distinkt separata arenor och aktörer problematiseras och istället ses som samkonstituerande parter i irreguljära relationer (Callon, 1999; Jasanoff, 2004; Shapin & Schaffer, 1989; Wehrens, 2014). Samproduktionsansatser (Jasanoff, 2004) betonar just att både samhälle och vetenskap är sociala aktiviteter producerade av aktörer (Knorr-Cetina, 1981; Latour & Woolgar, 1979), att samproduktion sker i maktstrukturer (Harding, 1991), samt omöjligheten att studera vetenskap och samhälle – eller kunskap och policy för den delen – som olika processer.

Förankringen i demokrati respektive folkhälsa kan därför vidare förstås som så kallade *gränsövertygelser* (boundary beliefs, Winter, 2019).^4^ Gränsterminologin belyser överbryggande interaktion mellan olika aktörer och arenor. Genom att samlas runt löst definierade men ändå sammanhängande övertygelser, begrepp (Löwy, 1992) eller objekt (Star & Griesemer, 1989) som tillåter en tolkningsflexibilitet (interpretive flexibility, Star & Griesemer, 1989), kan aktörer kommunicera utifrån sina respektive positioner över discipliner, ideologier eller andra skiljelinjer. Repetition är en avgörande kraftfull praktik i denna interaktion då den i specifika kontexter både etablerar kunskapsanspråk och stärker positioner medan andra försvagas (Winter, 2019). Här har Timbro varit föredömligt repetitivt i sitt arbete att utmana den svenska alkoholpolitiken utifrån en omtolkning av folkhälsoarbetet.

Vad syftar samproduktion till annat än att beskriva ett komplext nätverk av ord, aktiviteter och praktiker som lånas, repeteras och omformuleras? Det som kan saknas i sådana omfattande och samtidigt potentiellt platta teoretiska begrepp är en empirisk inblick i det som i komplex kunskapsproduktion ändå återkommande skapar struktur och mening, ger riktning och konsekvenser för produktionen. Som vi har försökt visa är användningen av demokratin och folkhälsan relativt godtycklig men eftersom de både som begrepp och politiska övertygelser är tillräckligt öppna kan aktörerna både samspela med befintliga praktiker av demokrati (exempelvis kvinnors rösträtt) och folkhälsa (exempelvis för specifika grupper medborgare) samt fylla dem med ny mening som passar argumenten. Ord är trots allt bara ord, men de blir livsviktiga när de fylls av handling och konsekvens.

Vad förskjutningen från demokratiideal till hälsoideal har för konsekvenser är inte självklart. Folkhälsomotiv är inte längre nödvändigtvis kopplat till en viss sorts restriktiv politik och samhället verkar ha rört sig mot en konvergens avseende den gemensamma ambitionen att prioritera folkhälsomål, men nya definitioner av målen tillkommer och en annan (liberal) väg föreslås för att nå dem. Å ena sidan bäddar detta för en potentiell utveckling av ett bredare folkhälsopolitiskt engagemang. Å andra sidan vittnar tidigare iakttagelser om uppluckring av traditionella välfärdsideal om en klar problematik gällande vad som kan anses vara de viktigaste folkhälsoprioriteringarna (se t.ex. Dahlgren, 2008; 2014). Det är sammanfattningsvis rimligt att se detta som ett slags appropriering av ord som möjliggör handlingar långt ifrån konkreta folkhälsofrämjande förslag.

## Notes

1. Det hävdas ibland felaktigt att nej-sidan vann med minsta möjliga marginal. Det stämmer visserligen att det var jämnt – 50,8 procent nej mot 48,8 procent ja – men det var heller aldrig meningen att införa ett alkoholförbud utifrån enkel majoritet.

2. Det bör understrykas att vi enbart intresserar oss för oppositionella kunskapsaktörer här.

3. Totalkonsumtionsmodellen är en vidareutveckling av demografen Sully Ledermans studier på 1940-talet. Den vilar på premissen att den genomsnittliga per capitakonsumtionen är vägledande för antalet storkonsumenter, något som har varit vägledande för svensk alkoholpolitik för att exempelvis genomföra åtgärder rörande tillgänglighet och pris (Bruun, Edwards, Lumio, Mäkelä, Pan, Popham, Room, Schmidt, Skog, Sulkunen & Österberg, 1975; Svensson och Enefalk, 2012).

4. Se också Löwy (2009) om boundary concepts/gränsbegrepp och Star och Griesemer (1989) om boundary objects/gränsobjekt.

## Appendix 1. Förteckning Timbromaterialet

[Table table1-14550725211072631]

**Table table1-14550725211072631:**

Nr.	Datum	Titel
T1	120619	Alkoholen en lyckad svensk liberalisering
T2	160331	Förbudsministern
T3	160429	Tänk på procenten: Systembolagets ogrundade kampanj
T4	160722	Farväl till Systembolaget
T5	170330	Du blir aldrig vuxen i folkhälso-Sverige
T6	170428	Systembolaget borde vara mer som public service – och vice versa
T7	170430	Nykterhetsrörelsen gör livet svårt för nykterister
T8	170504	I skuggan av systembolaget
T9	170510	Folkhälsopolitiken handlar om makthunger
T10	170510	Nanny state index 2017
T11	170621	Hellre öl i mataffären än alkoglass på systembolaget
T12	170629	Ofullständiga rättigheter: hur restaurangägare drabbas av kommunalt godtycke
T13	170705	En nykterhetslobbyists världsbild
T14	170719	Serveringstillståndet är förlegat
T15	170815	Våga ta ställning mot monopolet
T16	171003	Systembolaget lobbade på centerstämman
T17	171108	Förr eller senare går barnvaktstaten för långt
T18	171122	Gräset är grönare för den som lider av kronisk smärta
T19	171221	Fira inte en vit jul
T20	180402	Låt inte Miljöpartiet göra Swedavia till sin megafon
T21	180507	Jubla inte över gårdsförsäljningen
T22	180724	Alkoholskatt för finansministerns skull
T23	181023	Polisen ser bara knarkets baksidor
T24	181221	Julen har allt de progressiva hatar
T25	190115	Förmynderireflexen slår till igen
T26	190218	Ett avdrag är inte ett bidrag
T27	190219	Reklamen och moralpaniken
T28	190327	Myten om den otillgängliga primärvården
T29	190415	Kampen mot livsnjutarna
T30	190430	Hoten mot siste april
T31	190430	Nanny state index 2019
T32	190514	Borgerligheten borde inte låta sig utklassas av frp
T33	190520	Svensk alkoholpolitik – en gränslös fars
T34	190618	Sälj inte produkter ni inte står för
T35	190701	Ta nästa steg – förbjud pälsdjur på uteserveringar
T36	190703	Fmsf: Sverige har blivit mindre fritt
T37	190801	Sjöfyllerilagen är nykterhetspopulism
T38	190808	Rökförbudet skadar fler än det hjälper
T39	191116	Alkoholmonopolet är misslyckad politik
T40	191119	Så spelar S ut liberaler och konservativa mot varandra
T41	200102	Djävulen bor i detaljerna
T42	200223	Systembolagssamhället
T43	200331	Folk borde få köpa hem en corona från krogen

## References

[bibr1-14550725211072631] Allern S. Pollack E. (2016). Swedish Advocacy think tanks as new sources and agenda setters. Politik, 1(19), 61–76. 10.7146/politik.v19i1.27395

[bibr2-14550725211072631] Ahrne G. Brunsson N. (2011). Organization outside organizations. The significance of partial organization. Organization, 18(1), 83–104. 10.1177/1350508410376256

[bibr3-14550725211072631] Arnoldi J. (2007). Universities and the public recognition of expertise. Minerva, 45, 49–61. 10.1007/s11024-006-9028-5

[bibr4-14550725211072631] Babor T. F. (2009). Alcohol research and the alcoholic beverage industry: Issues, concerns and conflicts of interest. Addiction, 104(suppl 1), 34–47. 22. 10.1111/j.1360-0443.2008.02433.x 19133913

[bibr5-14550725211072631] Bengtsson Å. kvinnor N. *Folkbildare, företagare och politiska aktörer*. *Vita bandet 1900–1930*, Göteborg 2011.

[bibr6-14550725211072631] Benner M. Widmalm S. (2011). Kunskap. Liber.

[bibr7-14550725211072631] Brownson R. C. Royer C. Ewing R. McBride T. D. (2006). Researchers and policymakers: Travelers in parallel universes. American Journal of Preventive Medicine, 30(2), 164–172. 10.1016/j.amepre.2005.10.004 16459216

[bibr8-14550725211072631] Bruun K. Edwards G. Lumio M. Mäkelä K. Pan L. Popham R. E. Room R. Schmidt W. Skog O.-J. Sulkunen Österberg E. (1975). Alcohol control policies in public health perspective (Helsinki, Finnish foundation for alcohol studies). WHO Europe/Addiction Research Foundation.

[bibr9-14550725211072631] Callon M. (1999). The role of Lay people in the production and dissemination of scientific knowledge. Science, Technology and Society, 4(1), 81–94. 10.1177/097172189900400106

[bibr10-14550725211072631] Carolan M. S. (2006). Sustainable agriculture, science and the co-production of ‘expert’ knowledge: The value of interactional expertise. Local Environment, 11(4), 421–431. 10.1080/13549830600785571

[bibr11-14550725211072631] Collins H. M. Evans R. (2002). The third wave of science studies: Studies of expertise and experience. Social Studies of Science, 32(2), 235–296. 10.1177/0306312702032002003

[bibr13-14550725211072631] Dahlgren G. (2008). Neoliberal reforms in Swedish primary health care: For whom and for what purpose? International Journal of Health Services, 38(4), 697–715. 10.2190/HS.38.4.g 19069288

[bibr14-14550725211072631] Dahlgren G. (2014). Why public health services? Experiences from profit-driven health care reforms in Sweden. International Journal of Health Services, 44(3), 507–524. 10.2190/HS.44.3.e 25618987

[bibr15-14550725211072631] Denham A. Garnett M. (1996). The nature and impact of think tanks in contemporary britain. In Kandiah M. D. Seldon A. (Eds.), Ideas and think tanks in contemporary britain (Vol. I, pp. 43–61). Frank Cass.

[bibr73-14550725211072631] Edman J . (2015) “Temperance and modernity: alcohol consumption as a collective problem 1885-1913”, Journal of Social History, 49(1), 2015.

[bibr74-14550725211072631] Edman J . (2016) “Transnational nationalism and idealistic science: the alcohol question between the wars”, Social History of Medicine, 29(3). 10.1093/shm/hkv138 PMC4966481 27482147

[bibr16-14550725211072631] Forssell G. *Front mot förbudet!*, Stockholm 1921.

[bibr17-14550725211072631] Garsten C. (2013). All about ties. Think tanks and the economy of connections. In Garsten C. Nyqvist A. (Eds.), Organizational anthropology. Doing ethnography in and among complex organizations (pp. 139–154). Pluto Press.

[bibr18-14550725211072631] Garsten C. Rothstein B. Svallfors S. (2015). Makt utan mandat. De policyprofessionella i svensk politik. Dialogos Förlag.

[bibr19-14550725211072631] Garsten C. Sörbom A. (2015). Values aligned. The organization of conflicting values within the world economic forum. In Alexius S. Tamm Hallström K. (Eds.), Configuring value conflict in markets (pp. 159–177). Edward Elgar.

[bibr81-14550725211072631] Gieryn T. F . (1995). Boundaries of science. Jasanoff I. S. Markle G.E. Petersen J.C. Pinch T. (Red.), Handbook of science and technology studies, Sage Publications, Thousand Oaks, 393–443.

[bibr20-14550725211072631] Graham I. D. Logan J. Harrison M. B. Straus S. E. Tetroe J. Caswell W. Robinson N. (2006). Lost in knowledge translation: Time for a map? Journal of continuing education in the health professions, 26(1), 13–24. 10.1002/chp.47 16557505

[bibr21-14550725211072631] Hansson P. A. “Vårt parti och nykterhetsfrågan”, *Tiden*, No. 7, 1930.

[bibr22-14550725211072631] Haralds H. Kungl. Kontrollstyrelsen 1915–1917. En förvaltnings-historisk studie med statsrättslig bakgrund, jämte några kommentarer ur moralisk synpunkt utg. af förbundet för medborgarfrihet, Stockholm 1917.

[bibr23-14550725211072631] Harding S. G. (1991). Whose science? Whose knowledge?: Thinking from women’s lives. Cornell Univ. Press.

[bibr25-14550725211072631] Hawkins B. McCambridge J. (2014). Industry actors, think tanks, and alcohol policy in the United Kingdom. American Journal of Public Health, 104(8), 1363–1369. 10.2105/AJPH.2013.301858 24922137 PMC4103247

[bibr26-14550725211072631] Hawkins B. Parkhurst J. (2016). The’good governance’of evidence in health policy. Evidence & policy: a journal of research, debate and practice, 12(4), 575–592. 10.1332/174426415X14430058455412

[bibr27-14550725211072631] Heclo H. (1974). Modern social politics in britain and Sweden. From relief to income maintenance. Yale University Press.

[bibr28-14550725211072631] Hirdman Y. (1989). Att lägga livet tillrätta. Studier i svensk folkhemspolitik. Carlssons.

[bibr30-14550725211072631] Innvaer S. Vist G. Trommald M. Oxman A. (2002). Health policy-makers’ perceptions of their use of evidence: A systematic review. Health Services Research & Policy, 7(4), 239–244. 10.1258/135581902320432778 12425783

[bibr31-14550725211072631] Jasanoff S. (red.) (2004). States of knowledge: The co-production of science and the social order. Routledge.

[bibr32-14550725211072631] Jasanoff S. (2011). Constitutional moments in governing science and technology. Science & Engineering Ethics, 17, 621–638. 10.1007/s11948-011-9302-2 21879357

[bibr80-14550725211072631] Johansen KA Vandenbroeck M Vandevelde S . On the biopolitics of humane drug policies: What can we learn from 19th century sobriety boards? Nordic Studies on Alcohol and Drugs. 2021;38(5):498–516. 10.1177/14550725211015847 PMC8900182 35308817

[bibr33-14550725211072631] Johansson L. (1997). Svenskarna och alkoholmonopolet – ett historiskt perspektiv. Nordisk alkohol- och narkotikatidskrift, 14(3), s. 139. https://journals.sagepub.com/doi/pdf/10.1177/145507259701400311

[bibr34-14550725211072631] Knorr-Cetina K. D. (1981). The manufacture of knowledge: An essay on the constructivist and contextual nature of science. Pergamon Press.

[bibr35-14550725211072631] Latour B. Woolgar S. (1979). Laboratory life : The social construction of scientific facts. Beverly Hills: Sage.

[bibr36-14550725211072631] Lavis J. N. Oxman A. D. Moynihan R. Paulsen E. J. (2008). Evidence-informed health policy 1: Synthesis of findings from a multi-method study of organizations that support the use of research evidence. Implementation Science, 3, 53. 10.1186/1748-5908-3-53 19091107 PMC2621242

[bibr37-14550725211072631] Lundstedt V. *Förbudsfrågan ur rättslig synpunkt. Genom lagstiftning bör rättskulturen upprätthållas och icke undergrävas*, Stockholm 1920.

[bibr38-14550725211072631] Lundin P .,, … (ed.),. (2010). Science for welfare and warfare. Technology and state initiative in cold War Sweden. Science History Publications.

[bibr79-14550725211072631] Lundqvist Å. Petersen K . (eds.) (2010). In experts we trust. Knowledge, politics and bureaucracy in Nordic welfare states. Odense: University Press of Southern Denmark.

[bibr40-14550725211072631] Löwy I. (1992). The strength of loose concepts—boundary concepts, federative experimental strategies and disciplinary growth: The case of immunology. History of Science, 30(4), 371–396. 10.1177/007327539203000402

[bibr41-14550725211072631] McCambridge J. Hawkins B. Holden C. (2013). Industry use of evidence to influence alcohol policy: A case study of submissions to the 2008 scottish government consultation. PLoS Medicine, 10(4). 10.1371/journal.pmed.1001431 PMC3635861 23630458

[bibr42-14550725211072631] Medvetz T. (2012). Think tanks in america. The University of Chicago Press.

[bibr43-14550725211072631] Mitton C. Adair C. E. Mckenzie E. Patten S. B. Perry B. W. (2007). Knowledge transfer and exchange: Review and synthesis of the literature. Milbank Quarterly, 85(4), 729–768. 10.1111/j.1468-0009.2007.00506.x 18070335 PMC2690353

[bibr78-14550725211072631] Norström T Miller T Holder H Österberg E Ramstedt M Rossow I Stockwell T . Potential consequences of replacing a retail alcohol monopoly with a private licence system: results from Sweden. Addiction. 2010 Dec;105(12):2113–9. doi: 10.1111/j.1360-0443.2010.03091.x. Epub 2010 Sep 1. PMID: 20809914. 20809914

[bibr44-14550725211072631] Oliver K. Innvaer S. Lorenc T. Woodman J. Thomas J. (2014). A systematic review of barriers to and facilitators of the use of evidence by policymakers. BMC Health Services Research, 14, 2. 10.1186/1472-6963-14-2 24383766 PMC3909454

[bibr45-14550725211072631] Osborne T. (2004). On mediators: Intellectuals and the ideas trade in the knowledge society. Economy and Society, 33(4), 430–447. 10.1080/0308514042000285224

[bibr46-14550725211072631] Petrén G. (1988). Minoritetsskydd i en modern författning. Svensk Juristtidning, 72.

[bibr47-14550725211072631] Rabeharisoa V. Callon M. (2004). Patients and scientists in French muscular dystrophy research. In Jasanoff S. (Ed.), States of knowledge. The Co-production of science and social order (pp. 142–160). Routledge.

[bibr48-14550725211072631] Ramstedt M. , (red). Boman U. Engdahl B. Sohlberg T. Svensson J. (2010). Tal om alkohol 2010: en statistisk årsrapport från Monitorprojektet.

[bibr49-14550725211072631] Rich A. (2001). The politics of expertise in congress and the news media. Social Science Quarterly, 82(3). 10.1111/0038-4941.00044

[bibr50-14550725211072631] Rich A. (2005). Think tanks, public policy, and the politics of expertise. Cambridge University Press.

[bibr51-14550725211072631] Sacks H. (1995). Lectures on conversation (New edition; G. Jefferson & E. A. Schegloff, Eds.). Wiley-Blackwell.

[bibr52-14550725211072631] Saldaña J. (2015). The coding manual for qualitative researchers. Thousand oaks. C A: Sage.

[bibr53-14550725211072631] Sandberg C. E. *Leder förbudet till folknykterhet? Föredrag hållet i Eskilstuna den 14 november 1916*, Stockholm 1917.

[bibr54-14550725211072631] Santesson C. G. Efter förbudsomröstningen: riktlinjer för L. F. U. F:s fortsatta verksamhet, Stockholm 1922.

[bibr55-14550725211072631] Shapin S. Schaffer S. (1989). Leviathan and the Air-pump: Hobbes. Boyle, and the Experimental Life. Princeton University Press.

[bibr56-14550725211072631] Smith J. A. (1991). The idea brokers: Think tanks and the rise of the New policy elite. The Free Press.

[bibr57-14550725211072631] Socialdemokratiska agitationskommittén [SAK]. Arbetarepartiet och alkoholfrågan. *Några upplysningar om Förbundet för medborgarfrihet*, Stockholm 1916.

[bibr58-14550725211072631] Star S. L. Griesemer J. R. (1989). Institutional ecology, translations and boundary objects: Amateurs and professionals in Berkeley’s museum of vertebrate zoology, 1907–39. Social Studies of Science, 19(3), 387–420. 10.1177/030631289019003001

[bibr59-14550725211072631] Steele J. E. (1995). Experts and the operational bias of television news: The case of the Persian gulf War. Journalism & Mass Communication Quarterly, 72(4), 799–812. 10.1177/107769909507200404

[bibr60-14550725211072631] Stenius K. Babor T. F. (2010). The alcohol industry and public interest science. Addiction, 105(2), 191–198. 23. 10.1111/j.1360-0443.2009.02688.x 19804465

[bibr61-14550725211072631] Stone D. (1996). Capturing the political imagination: Think tanks and the policy process. Frank Cass. 1996.

[bibr77-14550725211072631] Svallfors S . (2016). Out of the golden cage. PR and the career opportunities of policy professionals. Politics and Policy 44 (1), 56–73.

[bibr62-14550725211072631] Svensson J. Enefalk H. (2012). Totalkonsumtionsmodellen. *En kort beskrivning. I J. Storbjörk (Red)*, *Samhället och drogerna: Politik, konstruktioner och dilemman*, 63–70.

[bibr63-14550725211072631] Sörbom A. (2018). Från snack till organiserade nätverk: Om tankesmedjors arbete för att värva andra för sina idéer. Sociologisk Forskning, 55(2–3), 365–387. Hämtad från https://sociologiskforskning.se/sf/article/view/18197

[bibr64-14550725211072631] Teitz M. B. (2009). Analysis for public policy at the state and regional levels the role of think tanks. International Regional Science Review, 32(4), 480–494. 10.1177/0160017609341388

[bibr65-14550725211072631] Trolldal B. Leifman H. (2015). Hur mycket dricker svensken?: registrerad och oregistrerad alkoholkonsumtion 2001–2014. Centralförbundet för alkhohol-och narkotikaupplysning.

[bibr66-14550725211072631] Tyllström A. (2013). Legitimacy for sale. Constructing a market for PR consultancy. Uppsala University.

[bibr67-14550725211072631] Weaver R. K. (1989). The changing world of think tanks. Political Science and Politics, 22(3), 563–578. 10.1017/S104909650003105X

[bibr68-14550725211072631] Wehrens R. (2013). Beyond two communities. The co-production of research, policy and practice in collaborative public health settings.

[bibr69-14550725211072631] Wehrens R. (2014). Beyond two communities – from research utilization and knowledge translation to co-production? Public Health, 128(6), 545–551. 10.1016/j.puhe.2014.02.004 24854762

[bibr76-14550725211072631] Winter K . (2019). Experiences and expertise of codependency: Repetition, claim-coupling, and enthusiasm. Public Understanding of Science, 28(2), 146–160. 30074434 10.1177/0963662518792807

[bibr75-14550725211072631] Winter K . (2020) “I'll Look Into it!” Lubricants in Conversational Coproduction. Minerva 58, 285–307 (2020). 10.1007/s11024-020-09394-6

